# Pretreatment of the urethral mucosa at the tip of the prostate: a retrospective review in preventing stress urinary incontinence after thulium laser enucleation of the prostate

**DOI:** 10.3389/fsurg.2024.1305006

**Published:** 2024-08-12

**Authors:** Yunfeng Liao, Yuting Wu, Junrong Zou, Ruohui Huang, Wei Xia, Yuanhu Yuan, Rihai Xiao, Xiaoning Wang, Gengqing Wu, Xiaofeng Zou, Guoxi Zhang

**Affiliations:** Department of Urology, Institute of Urology, Gannan Medical University, First Affiliated Hospital of Gannan Medical University, Jiangxi, Ganzhou, China

**Keywords:** benign prostatic hyperplasia, enucleation, stress urinary incontinence, external urethral sphincter, prostate apex

## Abstract

**Objective:**

Explore the clinical application value of urethral mucosal pretreatment at the tip of the prostate in preventing stress urinary incontinence (SUI) after thulium laser enucleation of the prostate (ThuLEP).

**Methods:**

Eighty-seven patients with benign prostatic hyperplasia (BPH) treated with ThuLEP from June 2021 to December 2022 were divided into two groups. Of these, 42 patients (group A) underwent conventional ThuLEP and 45 patients (group B) were enucleated after pretreatment of the urethral mucosa. At the tip of the prostate, pretreatment of the urethral mucosa consisted of pushing the gland separately on both sides at the level of the verumontanum and cutting off the mucosa near the external urethral sphincter clockwise and counterclockwise. The perioperative and postoperative follow-up indicators [operation time, hemoglobin reduction, complications, Qmax, International Prostate Symptom Score (IPSS), quality of life (QoL), and post-void residual (PVR) volume] of the two groups of patients were collected and compared. All patients were followed up 1 month after surgery.

**Results:**

All 87 procedures were successfully completed. There was no significant difference in age and gland size between the two groups (*P* > 0.05). There was no significant difference between operating time and hemoglobin reduction in the two groups (*P* > 0.05). The Qmax, IPSS, QOL, and PVR volume were significantly improved postoperatively in both groups (*P* < 0.05). Temporary SUI occurred in both groups [12 cases (28.5%) in group A and 3 cases (6.7%) in group B (*P* < 0.05)]. There was no significant difference in the incidence of infection and urethral stricture between the two groups (*P* > 0.05).

**Conclusion:**

Pretreatment of the urethral mucosa before ThuLEP for BPH significantly reduces the incidence of SUI after surgery. This technique, which preconditions the apical urethral mucosa of the prostate, is safe and effective, has few complications, and is worthy of clinical application.

## Introduction

Benign prostatic hyperplasia (BPH) is a frequent pathology in people over 50 years old (1). It is a non-malignant growth of the prostate gland (2). Its clinical expression is characterized by lower urinary tract symptoms (LUTS) (3, 4). These symptoms alter patients’ quality of life (QoL) and cause complications that can destroy kidney function.

Endoscopic surgical management of BPH continues to evolve with technological advances (5–8). In 1998, this surgical management experienced an important turning point with the discovery of endoscopic enucleation of the prostate (9). This surgical technique offers several advantages. It allows the entire adenomatous prostatic block to be enucleated, while limiting blood loss during surgery and reducing the risk of perforating neighboring organs (10–12). Since the discovery and description of endoscopic enucleation of the prostate, several variations of this technique have been described (13). Despite all these improvements, researchers have increasingly reported the onset of stress urinary incontinence (SUI) after applying this technique for BPH (14–16).

According to the anatomy of the external urethral sphincter, it is assumed that the mucosa near the external urethral sphincter plays an important role in urinary control. Therefore, we introduce and describe this novel technique for pretreating the prostate apex before thulium laser enucleation of the prostate (ThuLEP).

## Material and methods

### Subjects

This was a monocentric retrospective observational study of patients with BPH treated with ThuLEP from June 2021 to December 2022. The study included 87 patients aged between 60 and 80 years old who were diagnosed with symptomatic BPH with a prostate volume of 50–80 ml. Patients with severe comorbidities, prostate cancer, neurogenic bladder, and severe urethral stricture were excluded. Patients were divided into two groups: 42 (group A) underwent conventional ThuLEP, and 45 patients (group B) had prostate apex pretreatment before ThuLEP. The baseline results of the two patient groups were compared after the surgical procedures. The incidence of SUI was investigated in each group of patients. All surgeries were conducted by a single surgeon who had previously performed 120 ThuLEP procedures on patients.

### Instruments and surgical procedures

The 2 μm thulium (Tm:YAG) laser (SRM-T125 surgical laser, Raykeen Laser Products, Shanghai, China) was used at two distinct energy levels: 87.5 W for incising the lateral margins of the median lobe and 37.5 W for coagulating capsule-perforating vessels during the blunt enucleation of the prostatic adenoma. Laser energy was delivered through a reusable 550 µm laser fiber (Raykeen Laser Products, China).

**Figure 1:**
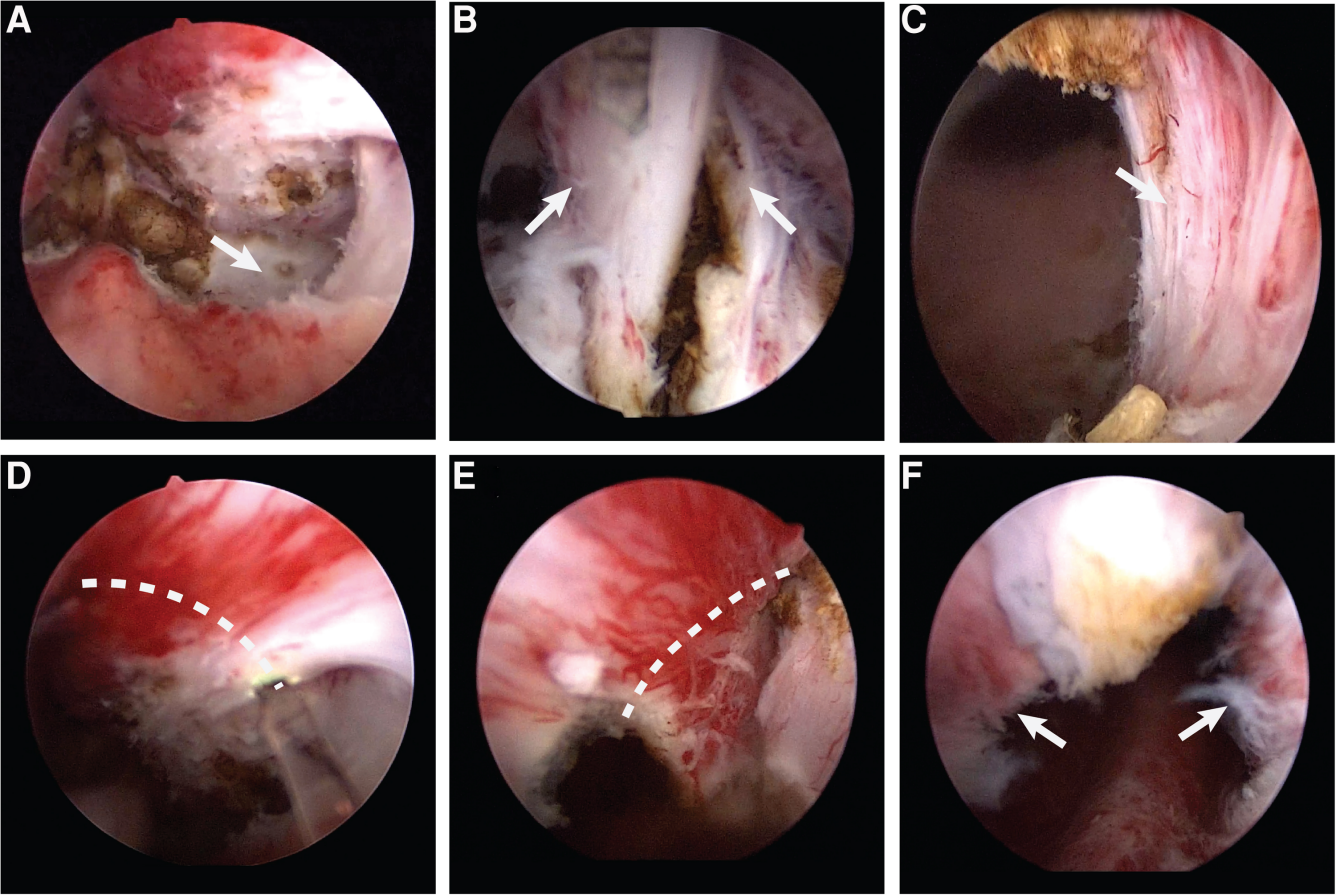
(**A**) The plane between the adenoma and the surgical capsule. (**B**) The 12 o'clock adenoma. (**C**) The exposed external urethral sphincter. (**D**) Make a incision along the dotted line counterclockwise. (**E**) Make a incision along the dotted line clockwise. (**F**) The preserved urethral mucosa at the prostatic apex.

Surgeries in group A were performed according to the conventional enucleation technique (17). The patients were placed in the dorsal lithotomy position under spinal anesthesia; preparation was then completed and sterile draping was applied. We used a 26F resectoscope, with continuous flow by normal saline. The camera was placed on the lens and the handpiece loosened such that the resectoscope could be rotated easily while holding the camera in a stationary position. Finally, the 550 µm laser fiber was inserted through the working channel of the resectoscope. Once the resectoscope was in the bladder, the ureteric orifices could be located, although a large median lobe made it difficult. The verumontanum and external sphincter were then observed. The prostate enucleation was performed from the tip of the prostate to the bladder neck. To begin the dissection, the beak of the resectoscope was guided toward the bilateral lobes through a transverse movement from the verumontanum; then, the plane between the adenoma and the surgical capsule developed (Figure 1A). The beak of the resectoscope was used to detach the left lobe along the surgical capsule from the 5 o’clock position of the prostatic apex to the 1 o’clock position of the bladder neck. The right lobe was detached along the surgical capsule from the 7 o’clock position of the prostatic apex to the 11 o’clock position of the bladder neck. Then, the 12 o’clock adenoma was severed (Figure 1B). The median lobe was detached after the dissection of the proximal adenoma of the verumontanum. The hyperplastic glands were enucleated *en bloc* and pushed into the bladder. Then, the optic was retracted to the external urethral sphincter for observation; the external urethral sphincter was found to be exposed (Figure 1C). The procedure continued with an inspection of the prostatic fossa to search for bleeding, which must have carefully coagulated. The procedure ended with the morcellation of the enucleated adenoma using a morcellator. A three-way catheter was placed at the conclusion, with continuous bladder irrigation.

Surgeries in group B were performed after pretreatment of the urethral mucosa. The preparation and installation of the patient followed the same procedure used for group A; the instruments used were also identical. At the tip of the prostate, pretreatment of the urethral mucosa consisted of pushing the gland separately on both sides, at the level of the verumontanum, to the surgical capsule. Then, the external urethral sphincter was exposed at the 5 and 7 o'clock positions, and the mucous membrane was broken clockwise and counterclockwise from that point to the 12 o'clock position (Figures 1D,E). Thus, the urethral mucosa at the tip of the prostate was preserved (Figure 1F), which filled the gap left by the contraction of the external sphincter after ThuLEP. After this pretreatment of the urethral mucosa at the tip of the prostate, enucleation could continue following the steps described above.

All patients were followed up 1 month after surgery. Careful history taking and physical examination in the clinic helped to diagnose SUI. The absence of stress incontinence was assessed by defining complete urinary control as no pad usage. If more than one pad was required for urinary control, the subject was considered positive for stress incontinence.

### Statistical analysis

Normality of the data was tested using a Kolmogorov–Smirnov test. Student’s t test and a Wilcoxon signed-rank test were used to analyze the differences in normally and non-normally distributed data between two groups, respectively. For paired preoperative and postoperative variance, two related sample Wilcoxon signed-rank tests were used to evaluate the differences in Qmax, the International Prostate Symptom Score (IPSS), the QoL score, and the post-void residual (PVR) volume. *P *< 0.05 was considered statically significant.

## Results

All 87 operations were successfully completed. The baseline characteristics were similar in each surgical group. There was no difference between the two groups with regard to demographics, prostate size, Qmax, the IPSS, QoL, and PVR volume (*P *> 0.05) (Table 1).

**Table 1:** Baseline characteristics of the patients (mean ± SD).

Group	A	B	*P*-value
No. of cases	42	45	
Age (years)	69.5 ± 6.1	70.1 ± 5.6	0.60
Prostate size (ml)	66.7 ± 9.0	67.0 ± 7.8	0.84
Qmax (ml/s)	6.7 ± 1.2	6.5 ± 1.0	0.56
IPSS	25.0 ± 4.1	24.6 ± 3.9	0.57
QoL score	4.6 ± 1.0	4.6 ± 0.9	0.90
PVR volume (ml)	69.8 ± 44.1	60.4 ± 44.8	0.33

*P* < 0.05 was considered signiﬁcant.

For the intraoperative data, there were no significant differences in operation time and decreases in hemoglobin (Hb) between the two groups (*P *> 0.05). For both groups of A and B, the operation times were 47.3 ± 10.4 and 49.8 ± 10.2 min, respectively (*P *= 0.27). The decreases in Hb were 15.5 ± 5.2 g/L and 15.6 ± 5.8 g/L, respectively (*P *= 0.89) (Table 2).

**Table 2:** Comparison of the intraoperative data (mean ± SD).

Group	A	B	*P*-value
Operation time	47.3 ± 10.4	49.8 ± 10.2	0.27
Decreases in Hb	15.5 ± 5.2	15.6 ± 5.8	0.89

*P* < 0.05 was considered signiﬁcant.

One month after surgery, we evaluated Qmax, the IPSS, QoL, and the PVR volume. Both groups showed a statistically significant improvement after surgery (*P *< 0.001) (Table 3). There were no significant differences between the groups with regard to postoperative Qmax, IPSS, QoL, and PVR volume (*P *> 0.05). The comparison of postoperative complications was not statistically significant for postoperative urinary infection and urethral stricture in both groups (*P *> 0.05). The rate of immediate SUI (following catheter removal) was 28.5% for group A, and 6.7% for group B, which was significantly different (*P *= 0.007) (Table 4).

**Table 3:** Comparison 1 month after surgery (mean ± SD).

Group	A	B	*P*-value
Urinary incontinence rate (%)	28.5	6.7	0.007
Urinary infection rate (%)	16.7	17.7	0.89
Urethral stricture (%)	7.1	8.9	0.77
Qmax (ml/s)	19.0 ± 3.3	18.7 ± 3.0	0.71
IPSS	5.0 ± 2.9	5.6 ± 3.0	0.46
QoL score	1.8 ± 1.0	1.9 ± 0.9	0.87
PVR volume (ml)	11.8 ± 9.6	14.1 ± 13.3	0.71

*P* < 0.05 was considered signiﬁcant.

**Table 4:** Comparison 1 month after surgery (mean ± SD).

Group	A	B	*P*-value
Urinary incontinence rate (%)	28.5	6.7	0.007
Urinary infection rate (%)	16.7	17.7	0.89
Urethral stricture (%)	7.1	8.9	0.77
Qmax (ml/s)	19.0 ± 3.3	18.7 ± 3.0	0.71
IPSS	5.0 ± 2.9	5.6 ± 3.0	0.46
QoL score	1.8 ± 1.0	1.9 ± 0.9	0.87
PVR volume (ml)	11.8 ± 9.6	14.1 ± 13.3	0.71

## Comment

The surgical treatment of BPH by prostatic enucleation offers many advantages, in particular the ablation of a large adenomatous volume and minimal intraoperative bleeding (11, 12). This is how this technique positions itself as the gold standard in the surgical management of BPH (10). Despite its performance, this procedure is frequently followed by postoperative SUI (18, 19). Several factors predisposing to the occurrence of SUI after prostatic enucleation have been described, such as age >70 years, a history of diabetes, a history of stroke, any previous LUTS/BPH medication use, a long enucleation time, and important intraoperative blood loss (14, 15, 18). Despite the absence of predisposing factors, the incidence of UI after prostatic enucleation remains high.

Types of UI include SUI, urge urinary incontinence (UUI), and mixed urinary incontinence (MUI). UUI is caused by impaired bladder function, whereas SUI is caused by injury to the external sphincter of the urethra. The procedural improvements we have made aim to reduce damage to the external sphincter of the urethra, thereby lowering the occurrence of postoperative stress incontinence. Therefore, we primarily evaluated SUI; UUI was not statistically analyzed.

Our study evaluates pretreatment of the urethral mucosa prior to prostatic enucleation for BPH. This evaluation will only be effective if we have a good understanding of urethral sphincter anatomy. The external urethral sphincter complex is found below the prostate apex; it is independent of the pelvic floor musculature but has a close relationship with it. It is innervated by autonomic branches of the pelvic plexus; the fibers of this plexus enter the urethral sphincter posterolaterally from both sides, mainly at the 5 o’clock and 7 o’clock positions and the 3 o’clock and 9 o’clock positions (20). The external urethral sphincter complex is made up of the outer striated muscle fibers and an inner muscle layer of the urethral sphincter, which consists of smooth muscle fibers (outer circumferential and an inner longitudinally oriented layer) (20, 21). The smooth muscle layer has its proximal limits at the level of the verumontanum. The pelvic floor musculature and external urethral sphincter, through contractions, allow patients to control their urination after prostate surgery (21). The urethral wall also includes a mucous membrane, the urothelium, which covers the sphincter musculature. The urothelium is plurilayered and leans back on a fibroelastic corium, which contains small blood vessels and lymphatics. The striated sphincter, smooth sphincter, and urethral mucosa together form the external sphincter urinary continence system. We envisage that the abovementioned urinary continence system should be preserved as completely as possible to improve urinary continence after surgery. During conventional prostatic enucleation, a part of the urethral mucosa, including the inner layer of smooth muscle at the distal end of the verumontanum, is stripped off by the endoscope, resulting in damage to a part of the external sphincter continence system. Therefore, we improved the conventional enucleation of the prostate and preserved the distal urethral mucosa and smooth sphincter with the verumontanum as the boundary. We believe that under the combined action of this part of the tissue, the urinary continence system can be well packed when the striated muscle is contracted and thus achieve good urinary continence. We assume that the mucosa near the external sphincter has the effect of filling the gap left by the contraction of the sphincter and thus plays an important role in urine control. Based on the anatomy described above, we believe that pretreatment of the urethral mucosa at the tip of the prostate could bypass the deleterious effects generated by the abovementioned mechanisms and thus preserve SUI after prostatic enucleation for BPH.

Pretreatment of the urethral mucosa at the tip of the prostate before prostatic enucleation performed in our study preserves the mucosa near the external sphincter. Thus, it fills the gap left by the contraction of the sphincter. This surgical procedure significantly reduces the incidence of SUI after surgery, from 28.5% to 6.7% (*P* < 0.05). These results are comparable with those found by Endo et al. (from 25.2% to 2.7%; *P* < 0.05), who used a similar technique in their study (13). This method is anterograde enucleation (enucleation from the bladder neck to the apex of the prostate), and finally the urethral mucosa is severed at the apex of the prostate, which also protects the external sphincter urinary control system well. However, this method requires an incision of the mucosa and gland at 12 o'clock at the apex of the prostate to look for the surgical capsule and then to further peel off the gland. The operation is complicated, the learning curve is long, and it is difficult to master. We used retrograde enucleation. As long as the urethral mucosa near the external sphincter is severed before enucleation, the external sphincter urinary control system can be protected. It is easy to learn and does not increase the overall operation time. Ferrari et al. (22) reported the technique of green light laser enucleation of the prostate with early apical release. This procedure involves marking circumferential limits between the adenoma and sphincter using coagulation. The critical step in this method is to create a circular mark between the verumontanum and external urethral sphincter. As the adenoma often extends beyond the verumontanum, and the extent varies among different patients, this approach poses two challenges: (1) the technical difficulty of standardizing the creation of the circular mark, and (2) the tendency to leave residual adenoma at the apex, which can lead to regrowth and recurrence. Our approach involves pushing the gland separately on both sides at the level of the verumontanum, reaching the surgical capsule. Subsequently, the external urethral sphincter is exposed at the 5 and 7 o'clock positions. Using this exposed portion of the external urethral sphincter as a landmark, we perform clockwise and counterclockwise mucosal detachment from that point to the 12 o'clock position. This not only standardizes the technique but also minimizes the residual adenoma, thereby reducing the likelihood of recurrence. More spectacular results were published by Takiuchi et al., who used a technique in which partial glands at the apex of the prostate are preserved instead of the urethral mucosa alone; their study did not reveal any cases (0%) of postoperative SUI (23). However, this method is prone to recurrence in the long term, and there is a possibility of the recurrence of urinary retention. In addition, prostatic enucleation by open surgery seems to provide good results in terms of postoperative continence. Helfand et al. reported SUI of 3.7% after surgery (24), and Serretta et al. reported 5.4% (25). Knowing the external sphincter urinary continence system, we can understand why the incidence of urinary incontinence is low after open prostatectomy. However, prostatic enucleation by open surgery does not promote Enhanced Recovery After Surgery (ERAS) principles and is invasive; therefore, it is now considered obsolete.

Pretreatment of the urethral mucosa at the tip of the prostate before prostatic enucleation improves QoL and the urine maximum flow rate. It does not increase the prevalence of infection, fever, and urethral stricture after surgery. It improves patient and surgeon satisfaction and could generate savings for administrations by avoiding additional costs related to the management of SUI occurring after surgery.

There are limitations to this study. The sample size was small due to the careful selection of patients. In addition, it was a retrospective study, and the results can be considered hypothesis-generating rather than definitive. Further research is needed to validate our findings. Therefore, a prospective study with a larger sample size is still required to confirm the feasibility of the modified procedure, which offers an improved surgical option for clinical quality BPH.

## Conclusions

Our study provides initial validation for the pretreatment of the urethral mucosa at the tip of the prostate technique, demonstrating a significant reduction in the incidence of post-surgical SUI. The approach is deemed safe and effective, showing low complication rates. However, owing to certain limitations in the study, further research is necessary to confirm these findings.

## Data Availability

The original contributions presented in the study are included in the article/Supplementary Material; further inquiries can be directed to the corresponding author.
